# Isolation of Quercetin from *Rubus fruticosus*, Their Concentration through NF/RO Membranes, and Recovery through Carbon Nanocomposite. A Pilot Plant Study

**DOI:** 10.1155/2020/8216435

**Published:** 2020-03-19

**Authors:** Muhammad Zahoor, Abdul Bari Shah, Sumaira Naz, Riaz Ullah, Ahmed Bari, Hafiz Majid Mahmood

**Affiliations:** ^1^Department of Biochemistry, University of Malakand, Chakdara Dir Lower, 18800 KPK, Pakistan; ^2^Medicinal, Aromatic and Poisonous Plants Research Centre (MAPRC), College of Pharmacy, King Saud University, PO box 2457, Riyadh 11451, Saudi Arabia; ^3^Central Laboratory College of Pharmacy King Saud University, PO box 2457, Riyadh 11451, Saudi Arabia; ^4^Department of Pharmacology, College of Pharmacy, King Saud University, PO box 2457, Riyadh 11451, Saudi Arabia

## Abstract

In this study, an attempt has been made to devise a method for a large-scale production of quercetin from a medicinal plant. The natural products are first isolated from plants and then synthesized commercially. During their synthesis, a number of impurities or side products are also formed, most of which are carcinogenic. Plant products have limited side effects. Therefore, they are considered safe to be used for systemic uses. In the *Rubus fruticosus* fruit, the ethyl acetate extract was loaded to 50 optimized silica gel columns. The effluents of columns were passed through the membrane system for concentration. A 100% recovery was achieved from the drain pipe in case of reverse osmosis membrane when the specified rely of the pilot plant was set on 25% rejection. About 95% recovery was achieved through the NF membrane while the 5% loss in permeate was recovered through magnetic carbon nanocomposite (characterized through a bar magnet, SEM, XRD, and EDX). The equilibrium time of adsorption was 83 min and followed by pseudo-first-order kinetics. The adsorption equilibrium data fitted well to the Langmuir isotherm model. Through the devised method, quercetin was successfully concentrated with high efficiencies; however, further studies are needed to validate the method.

## 1. Introduction

Plant materials are used throughout the world as home cure and as a raw material in the pharmaceutical industries [1]. Different techniques are used to isolate bioactive compounds from plants. This is a tedious job and usually, at the end of the process, one would get the required targeted compound in nanogram or milligram quantities [2–5]. After their structural elucidation through different spectroscopic techniques, they are then synthetically synthesized. For a large-scale production from plants, none of the researchers have tried to get them in high amounts from plants. There is a need for an efficient method to isolate the required bioactive compounds from plants in large quantities which will reduce the environmental problems associated with synthetic production. Also, the biomass is the renewable source of mass and energy so there would be no need of raw material supplementation. This is a pilot plant study, which in fact is an attempt to isolate bioactive compounds in large scale. Initially, in this study, we targeted quercetin. Quercetin is found in many plants, and due to this fact, we targeted its isolation [2–5].

Quercetin is one of the important bioflavonoids that exist in more than a hundred plants having anti-inflammatory, antihypertensive, vasodilator effect, antiobesity, antihypercholesterolemic and antiatherosclerotic activities [1–6]. It is used as a nutritional supplement against many diseases. The beneficial effects of quercetin are cardiovascular protection and anticancer, antitumor, antiulcer, antiallergy, antiviral, anti-inflammatory, antidiabetic, gastroprotective, antihypertensive, and immunomodulatory [7] effects. Quercetin and other polyphenolic compounds also have antibacterial potentials that has been confirmed by many researchers [8, 9].

Membrane technology is an emerging technology that are not only used in water purification but also used for the recovery of precious chemical substances from industrial effluents. Membranes have pores of molecular dimensions and substances having molecular weight higher than molecular weight cut off of the membrane could be retained from flowing through the membrane. Thus, they are used for recovery and concentration of certain chemical substances in industries [10]. An ultrafiltration membrane has large pores and they could not be used for such purposes. Although a nanofiltration membrane has small pores, still 100% recovery or concentration could not be achieved through such membranes. Reverse osmosis membranes on the other hand could effectively be used for 100% recovery of organic substances. However, energy consumption in reverse osmosis membrane operations is high which limits their uses [11].

To effectively use nanofiltration membranes, such types of operations are combined with adsorption processes. In such cases, adsorbents having a high surface area, if used, could effectively recover the amount of precious chemical that passed through the membrane into effluents. The most efficient adsorbents with a high surface area are activated carbon but their collection after use is a difficult job as their particles are very small and their settling time in reactors are longer. To remove this discrepancy, a number of researchers have attempted to make the activated carbon, magnetic [12–16]. The magnetic activated carbons on the one hand has a high surface area and on the other hand has a magnetic character. Thus, after use, it can easily be collected from the slurry through a magnetic process. The recovered adsorbent can be regenerated and the loaded precious compound can then be recovered [17–19].

The aim of this study was to devise a method for a large-scale production of quercetin from *Rubus fruticosus* plant.

## 2. Experimental

### 2.1. Extraction Protocol

Plant materials were extracted through methanol and were fractionated using various solvents. All the fractions were subjected to HPLC-UV analysis. The ethyl acetate fraction with a broad peak of quercetin was further subjected to silica gel column isolation. Initially, standard quercetin in pure form was passed through silica gel columns and its retention time and number of vial was noted. To optimize the column, the packing silica gel was subjected to HCl, NaOH (pH 5 to 14), and temperature treatments (amount of silica taken = 30 g). After complete absorption and dryness, it was loaded to the columns. The temperature treatment was done by keeping it in the oven for 24 h at 0, 50, 100, 150, 200, and 250°C. A column from which quercetin was released in a comparatively shorter time was the optimized one. About 50 optimized columns were then simultaneously operated for isolation of quercetin from *Rubus fruticosus*. The extract-loaded columns were eluted with n-hexane and ethyl acetate (5 to 30%) mixture fed to these columns through a peristalsis pump equipped with pipe and T joints for equal supply to each column. At the predetermined time intervals, the effluents of each column containing quercetin were collected in a common container. The purity of the isolated targeted compound was confirmed through HPLC (figures [Supplementary-material supplementary-material-1]). Further confirmation was carried out through carbon and proton NMR as shown in [Supplementary-material supplementary-material-1].

### 2.2. Concentration of Isolated Quercetin

Two membranes NF and RO were used in this study in order to concentrate quercetin present in the column effluents. The effluents were allowed to pass through the RO membrane in a crossflow manner where rely was fixed at 25% rejection. The targeted compound was collected from the drain of the membrane and was recycled three times. The remaining solvent was evaporated at room temperature. The energy consumption of RO membranes is high; therefore, the NF membrane system was used as its alternative. In a concentration through the NF membrane, 95% of isolated compound was present in the drain while 5% in permeate which was collected through magnetic carbon nanocomposite (MCN) prepared from biomass. The same procedure as described above for RO was used for NF membrane operation as well.

### 2.3. Preparation and Characterization of Adsorbent

MCN was prepared from a biomass precursor of pineapple. Small pieces of the precursor was dried and transferred to a container containing ferric chloride and ferrous sulphate. After some time, 5 mol/L (100 mL) NaOH was added to the mixture at 70°C and was then charred and ignited in the presence of N_2_ gas atmosphere for ten hours. The final product was washed with distilled water till neutral pH and kept in an electric oven till dry. Characterization of the prepared composite was done by XRD (with a nickel filter using monochromatic Cu K*α* rays, having a wavelength of 1.5418 Å), SEM (scanning electron microscope), and EDX (energy-dispersive X-ray).

### 2.4. Adsorption Studies of Quercetin

The adsorption equilibrium and kinetic parameters of quercetin on MCN were determined using known models and isotherms, in order to get 100% recovery of the 5% loss of quercetin in permeate in case of NF operations. A series of solutions from 5 to 50 ppm were prepared and 50 mL from each was contacted with 0.01 g of MCN. After shaking for 2 h, the adsorbent was removed from the solution through a bar magnet and the remaining concentration of quercetin in the solution was determined using UV/visible spectrophotometer. The data was then fitted in the Langmuir and Freundlich models to decide which one is the best model to describe the equilibrium data. In kinetic studies, 20 ppm solution was contacted with 0.01 g of MCN in a number of flasks and shaken for different intervals of time. The remaining concentration in each flask was determined and data was fitted into the pseudo-first- and second-order kinetic models to decide which of the model fitted the data well.

## 3. Results and Discussion

### 3.1. Optimization of Silica Gel Columns

First, the methanolic extract was subjected to fractionation. Amongst different fractions, broad HPLC peak of quercetin was observed for ethyl acetate fraction. The ethyl acetate extract was then loaded to a silica gel column. The column was eluted with n-hexane/ethyl acetate solvent system. In an untreated silica gel column, the maximum amount of quercetin was obtained in vial number 79. To enhance the release of quercetin from the column, the silica gel was subjected to acid, base, and temperature treatments. The elution of quercetin was enhanced by all these treatments. From temperature-treated silica gel column, quercetin was released in vial number 42, while in the base-treated column, it was released in a high amount in vial no. 56 and vial no. 74 in the case of the acid-treated column. Although temperature treatment caused early release of selected compound, a high amount of it was obtained from the acid-treated column. Thus, finally, acid and temperature treatments were combined to get a maximum yield (vial no 48).

The column diameter and height affect the release of a particular compound when it passed through it. It means that different columns would have different release times of a particular compound. About 50 columns were then optimized to get the maximum yield of the targeted compound.

### 3.2. Concentration of Quercetin

Ethyl acetate extract solution was fed to 50 optimized silica gel columns through a peristaltic pump. A stopwatch was turned on, and at a specified time, the effluents from each column were collected in a common container. After 4 operations, about 1 L of effluents containing quercetin was fed to a pilot plant comprising a multispeed pump, a pressure pump, monometers, flowmeters, and RO and NF membranes.

First, the effluents were subjected to the RO membrane. The membrane was operated in a crossflow mood and rely was set on 25% rejection. After four cycles, the volume of effluents containing selected compound was reduced to 100 mL. Natural products generally dissociates at high temperatures. If 1 L solution is subjected to high-temperature evaporation, definitely it will decompose into various components. The membrane operation has reduced its volume to 100 mL, and now, further concentration through heat will not cause such deterioration of the compound structure and even can be evaporated at room temperature.

The RO membrane operations are economically expensive as they need a high amount of energy as compared to NF membranes. To get the target with NF membranes, the 1 L effluents obtained from optimized columns was subjected to a pilot plant. However, this time, the NF membrane was installed instead of the RO membrane. About 95% recovery was achieved and 5% loss of selected compound was encountered. To get them back, the resulting permeate of the NF membrane was treated with 5% adsorbent. After shaking for about 80 minutes, the quercetin-loaded adsorbent was recovered through magnetic applications and was regenerated through acetic acid treatment. After treatment, adsorbent was separated from the slurry and quercetin was recovered from the acetic acid solution.

**Figure 1:**
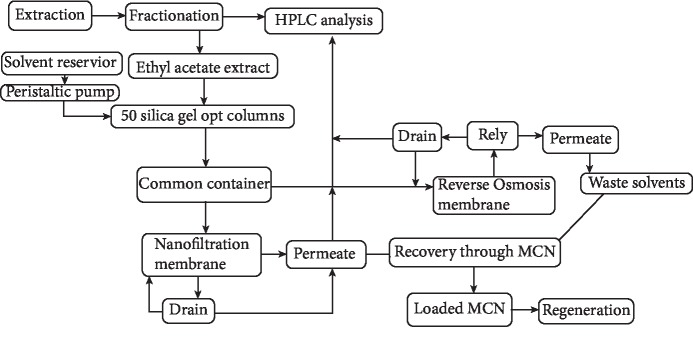
Schematic diagram of the study.

The schematic diagram of the whole process is given in Figure 1.

The detail adsorption of quercetin by magnetic carbon nanocomposites is given as follows.

### 3.3. Characterization of the Adsorbent

The adsorbent with a magnetic character was prepared from waste biomass. To confirm whether it has magnetic character or not, it was subjected to a bar magnet; all the particles stick to the magnet confirming that it has a magnetic character. Further, it was characterized by other instrumental techniques as described below.

[Supplementary-material supplementary-material-1], the energy-dispersive X-ray graph of MCN, shows the elemental composition of the adsorbent and confirmed the presence of iron. [Supplementary-material supplementary-material-1], the SEM images, shows that MCN particles are spherical with a size distribution of 45-65 nm. The white spots confirm the crystallization of the iron oxide over the adsorbent surface. The aggregates observed were lumps of adsorbent formed due to moisture contents present in the sample which decreases the surface area of MCN which consequently decreases its sorption capacity. The deposited iron oxide (Fe_3_O_4_) has the cubic crystalline structure as observed previously [17–20].

XRD is an important technique used for size distribution and crystal structure of solids [19]. [Supplementary-material supplementary-material-1] shows the presence of Fe_3_O_4_ deposited on a carbonaceous material which is evident from characteristic diffraction peaks of Fe_3_O_4_ at 2*θ* of 30, 35.7, 44, 53, 57.95, and 62.5° corresponding to indices of 220, 311, 400, 422, 511, and 400. These indices correspond to the cubic crystalline structure of magnetite [17–20].

### 3.4. Adsorption Isotherms

A number of adsorption models are used to describe the equilibrium data. Amongst them, the Langmuir and Freundlich models are most frequently used [21, 22].

**Figure 2:**
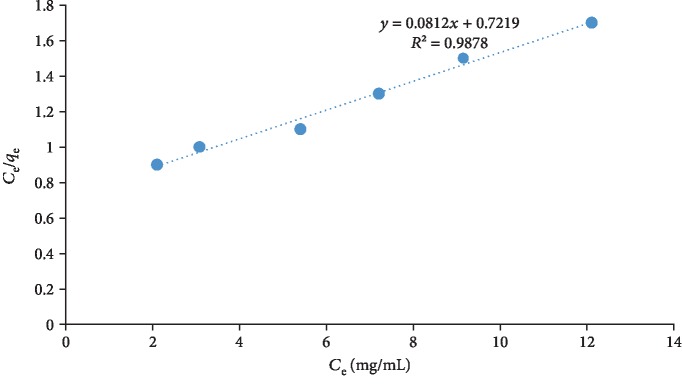
Langmuir isotherm for quercetin adsorption on MCN.

The mathematical representation of Langmuir isotherm can be given as follows:

(1) $$\begin{array}{c} \frac{{\text{C}}_{\text{e}}}{q_{\text{e}}}=\frac{1}{K_{L}q_{m}}+\frac{C_{\text{e}}}{q_{\text{m}}}, \end{array}$$

where *q*_e_ is the amount of quercetin adsorbed (mgg^−1^), *C*_e_ is the equilibrium concentration of the adsorbate (mgL^−1^), *q*_*m*_ (mg/g) and *K*_*L*_ (L/mg) are Langmuir constants related to maximum adsorption capacity and energy of adsorption, respectively. By plotting the specific adsorption (*C*_e_/*q*_e_) against the equilibrium concentration (*C*_e_), a curve presented in Figure 2 was obtained showing that adsorption obeys the Langmuir model. The values of *q*_*m*_ and *K*_*L*_ were calculated from the slope and intercept of the plots and were found to be 12.32 mg/g and 0.11 L/mg, respectively (Table 1).

**Figure 3:**
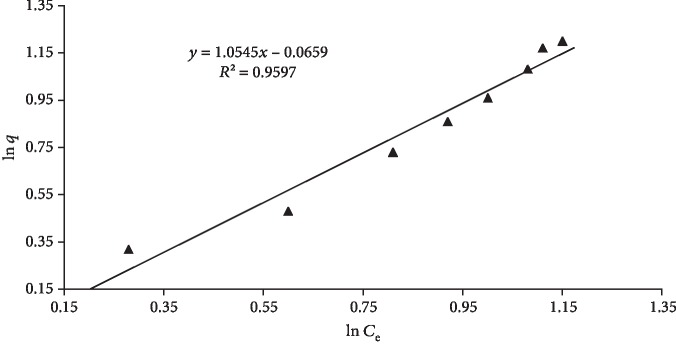
Freundlich isotherm for quercetin adsorption on MCN.

The Freundlich isotherm in its logarithmic form can be presented as follows:

(2) $$\begin{array}{c} \ln q_{\text{e}}=\ln k+\ln\frac{C_{\text{e}}}{n}, \end{array}$$

where *C*_e_ is the equilibrium concentration (mgL^−1^), *q*_e_ is the amount adsorbed (mgg^−1^), *k* and *n* are Freundlich constants related to the adsorption capacity and adsorption intensity, respectively. The values of the constants are presented in Table 1. *k* (1.07 mg/g) and *n* (0.94) were calculated from the slope and the intercept of the plot (Figure 3). Based on the regression constant values, the Langmuir model was found to be more appropriate to fit the adsorption equilibrium data well.

### 3.5. Adsorption Kinetics

**Figure 4:**
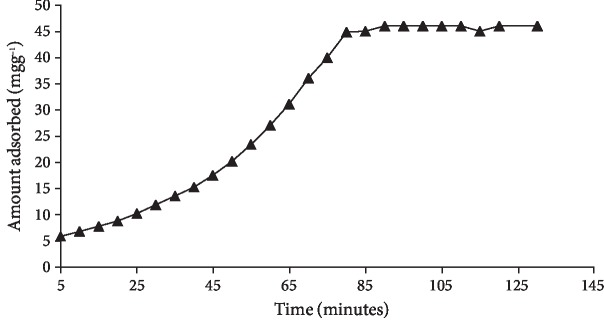
Effect of contact time on quercetin adsorption onto MCN.

The knowledge of adsorption kinetic is important as it gives information about the saturation point of the adsorbent: the maximum amount of adsorbate to be removed and in how much time. Contact time of adsorbent with adsorbate is usually determined to establish the equilibrium time. For this purpose, 20 ppm solution of quercetin was contacted with MCN (Figure 4) for 140 min. The saturation of adsorbate took place after a time interval of 83 min which was the equilibrium time of adsorption.

**Figure 5:**
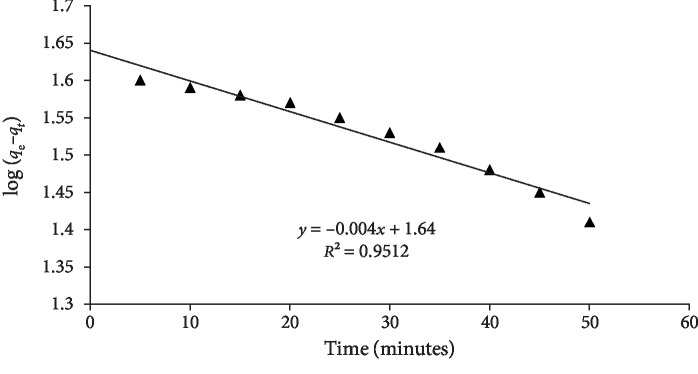
Pseudo-first-order kinetic model of quercetin adsorption on MCN.

Pseudo-first- and second-order kinetic models were used to establish kinetic parameters of adsorption. The mathematical forms of these models can be presented as follows:

(3) $$\begin{array}{c} \ln\left(q_{\text{e}}-q_{t}\right)=\ln q_{\text{e}}-K_{1}t, \end{array}$$

(4) $$\begin{array}{c} \frac{t}{q_{t}}=\frac{1}{K_{2}}+\frac{t}{q_{\text{e}}}, \end{array}$$

where, in Equation (3), *q*_*t*_ (mg/g) is the amount adsorbed at time *t*, while, *q*_e_ (mg/g) is the amount adsorbed at equilibrium time, *K*_1_ (min^−1^) is the rate constant of the pseudo-1^st^-order kinetics. The values of *q* and *K*_1_ (min^−1^) were calculated from the slope and intercept of the plot obtained from plotting ln(*q*_e_ − *q*_*t*_) vs. *t* (Figure 5). The values of *K*_1_ and *q*_e_ were found to be 0.0041 min^−1^ and 5.16 mg/g, respectively (Table 2).

**Figure 6:**
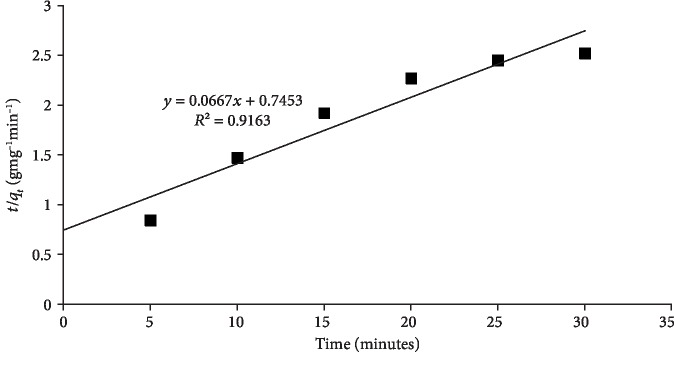
Pseudo-second-order kinetic model of quercetin adsorption on MCN.

While, in Equation (4), *K*_2_ (gmg^−1^ min^−1^) is the rate constant of adsorption for pseudo-2^nd^-order kinetics, *q*_e_ (mgg^−1^) is the amount of quercetin adsorbed at the equilibrium, and *q*_*t*_ is the amount of quercetin adsorbed at time *t*. The values of *K*_2_ (1.34 gmg^−1^ min^−1^) and *q*_e_ (14.99 mgg^−1^) were calculated from the slope and intercept of the plot by plotting *t*/*q*_*t*_ against *t* (Figure 6) and are presented in Table 2. Based on *R*^2^ values, best fit was obtained with the pseudo-second-order kinetic model.

## 4. Conclusion

The study was designed to devise a method for a large-scale production of bioactive compounds from plant origin as synthetic compounds are associated with a number of other complications. The idea of column chromatography, membrane separation, and adsorption were combined in the form of a pilot plant for achieving the goal. A number columns were first optimized to reduce the loss of solvents. Acid, base, and temperature treatments were used to optimize them. Organic compounds usually dissociated if high temperature is used to concentrate them. Rotary evaporators are used for their concentration which takes much time. The RO membrane system was used for the concentration of the isolated quercetin. A 100% recovery was obtained when the membrane plant was operated at 25% rejection. The use of an RO membrane is economically expensive; thus, to make the devised plant economic, RO membrane was replaced by NF membrane through which 95% recovery was achieved. To get back the 5% loss, magnetic activated carbon was used as an adsorbent. Although activated carbon has a high surface area, it needs a longer duration of time for setting to separate it after use from slurry; thus, MCN was used as it has a magnetic character that has been confirmed through bar magnet, XRD, SEM, and EDX studies and thus can be collected after use through a magnet within a few seconds. The adsorption parameters were also determined and the Langmuir model was found to explain the adsorption equilibrium data best, while the pseudo-first-order model fitted the kinetic data well.

## Figures and Tables

**Table 1:** Isotherm parameters of quercetin adsorption on nanocomposites.

Isotherm	Parameter	Value
Langmuir	*K*_*L*_ (L/mg)	0.11
	*q*_*m*_ (mg/g)	12.32
	*R* ^2^	0.9878

Freundlich	*k* (mg/g) (L/mg)^1/n^	1.07
	*n*	0.94
	*R* ^2^	0.9597

**Table 2:** Kinetic parameters of quercetin adsorption on nanocomposites.

Concentration (ppm)	Kinetic models	Values
20	Pseudo 1^st^ order	
	*K*_1_ (min^−1^)	0.0041
	*q*_e_ (mg/g)	5.16
	*R* ^2^	0.9512

20	Pseudo 2^nd^ order	
	*K*_2_ (gmg^−1^ min^−1^)	1.34
	*q*_e_ (mg/g)	14.99
	*R* ^2^	0.9163

## Data Availability

All the data associated with this research has been presented in this paper.

## Supplementary Materials

Supplementary Materials Figure 1S: HPLC chromatogram of Rubus fruticosus ethyl acetate fraction (peak at 10.448 represents quercetin). Figure 2S: HPLC chromatogram of Rubus fruticosus isolated quercetin C13 and H1 NMR spectra of quercetin. Figure 3S: HNMR of quercetin in DMSO. Figure 4S: expanded HNMR of quercetin in DMSO. Figure 5S: carbon13 NMR of quercetin in DMSO. Figure 6S: characterization of MCN (a) XRD, (b) SEM, and (c) EDX.
