# Supplementary material for: Isolation of Quercetin from Rubus fruticosus, Their Concentration through NF/RO Membranes, and Recovery through Carbon Nanocomposite. A Pilot Plant Study
Source: Biomed Res Int. 2020 Mar 19;2020:8216435. doi: 10.1155/2020/8216435 (PMC7109554; doi:10.1155/2020/8216435)
Supplement: Supplementary Materials — Figure 1S: HPLC chromatogram of Rubus fruticosus ethyl acetate fraction (peak at 10.448 represents quercetin). Figure 2S: HPLC chromatogram of Rubus fruticosus isolated quercetin C13 and H1 NMR spectra of quercetin. Figure 3S: HNMR of quercetin in DMSO. Figure 4S: expanded HNMR of quercetin in DMSO. Figure 5S: carbon13 NMR of quercetin in DMSO. Figure 6S: characterization of MCN (a) XRD, (b) SEM, and (c) EDX. [file 8216435.f1.docx]

**Supporting Information**


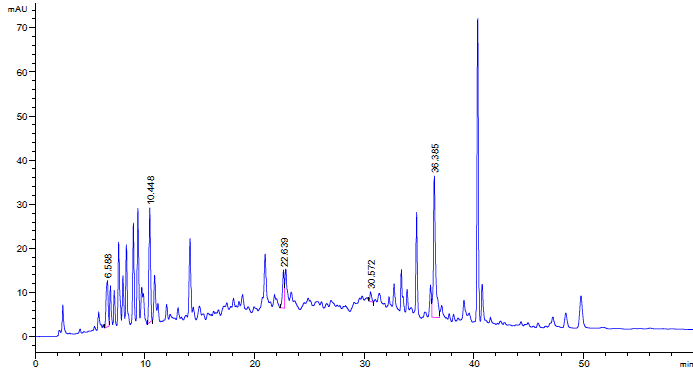


**Figure 1S:** HPLC chromatogram of *Rubus fruticosus* ethyl acetate fraction (peak at 10.448 represents quercetin)


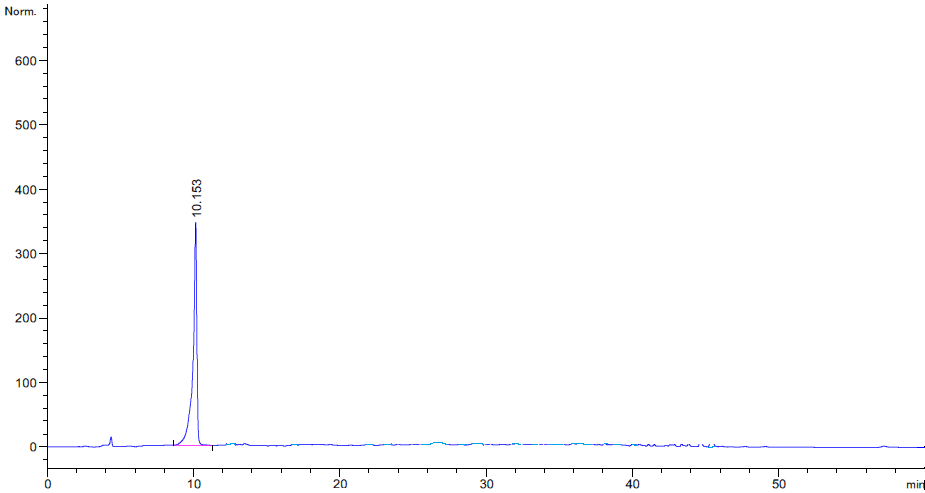


**Figure 2S:** HPLC chromatogram of *Rubus fruticosus* isolated quercetin

C^13^ and H^1^ NMR Spectra of quercetin


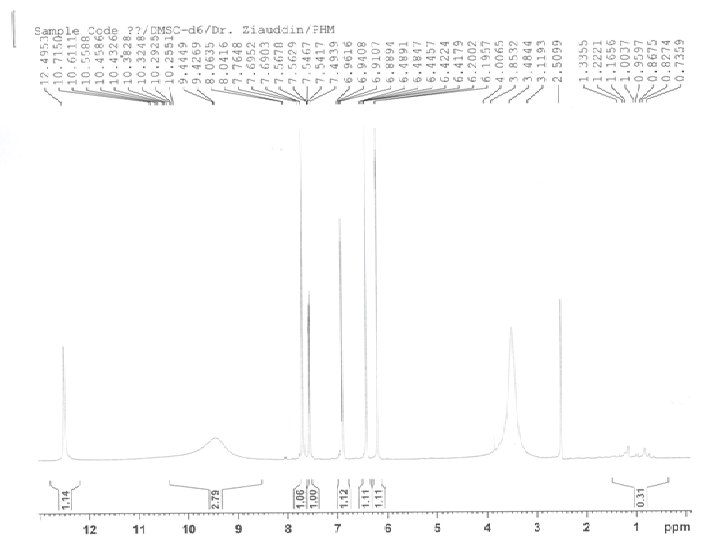


**Figure 3S:** HNMR of quercetin in DMSO


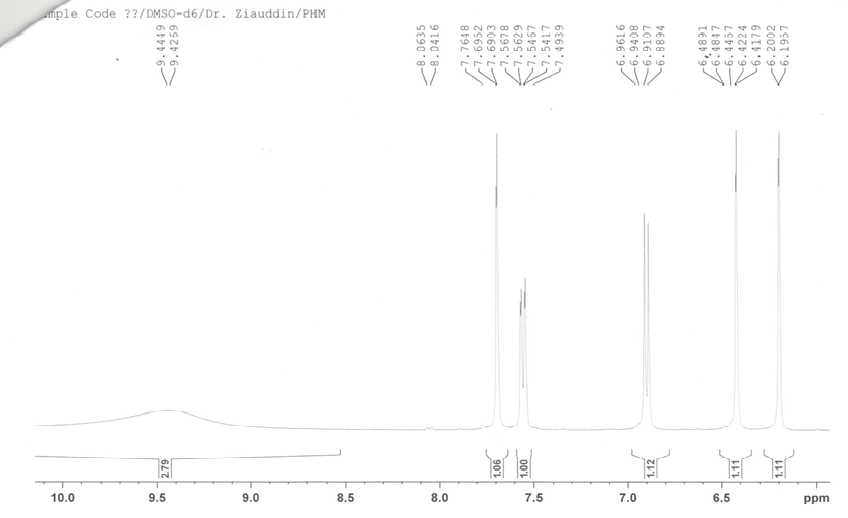


**Figure 4S:** Expanded HNMR of quercetin in DMSO


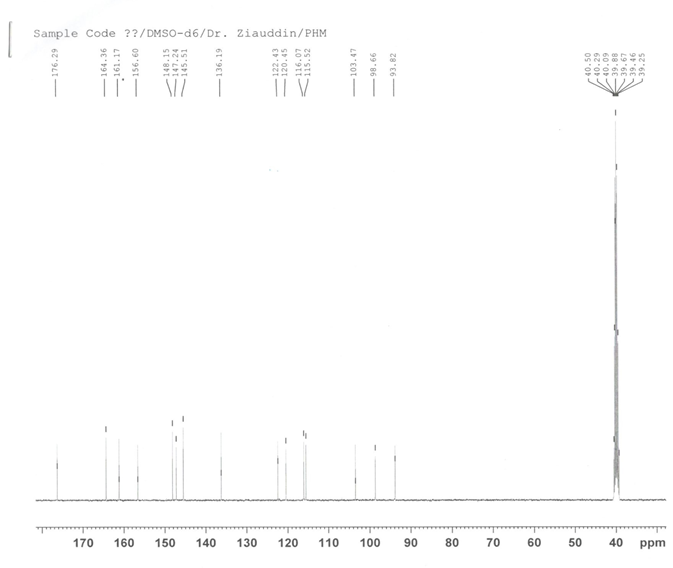


**Figure 5S:** Carbon^13^ NMR of Quercetin in DMSO

| 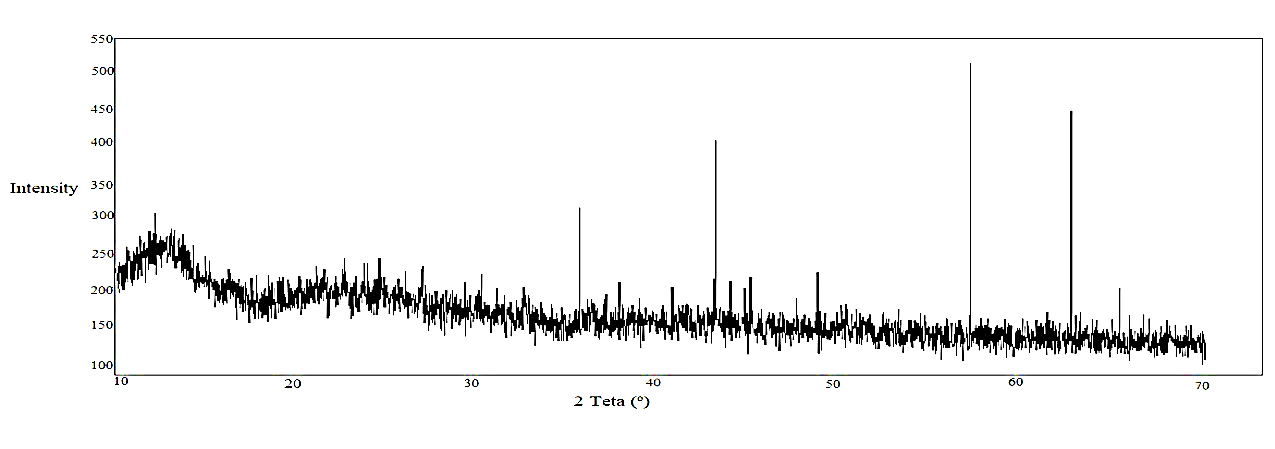a | 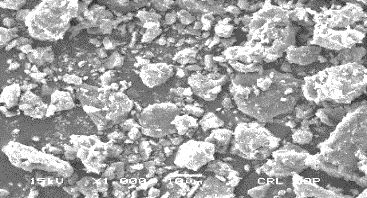  b |
| --- | --- |
| 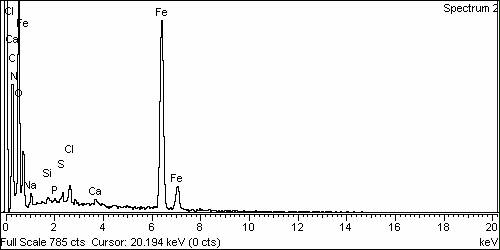  c | |

**Figure 6S:** Characterization of MCN a. XRD, b. SEM, c. EDX
